# Molecular Analysis of Full-Length VP2 of Canine Parvovirus Reveals Antigenic Drift in CPV-2b and CPV-2c Variants in Central Chile

**DOI:** 10.3390/ani11082387

**Published:** 2021-08-12

**Authors:** Véliz-Ahumada Alexis, Vidal Sonia, Siel Daniela, Guzmán Miguel, Hardman Timothy, Farias Valentina, Lapierre Lisette, Sáenz Leonardo

**Affiliations:** 1 Facultad de Ciencias Veterinarias y Pecuarias, Universidad de Chile, Santa Rosa 11735, La Pintana, Santiago 8820808, Chile; alexis.veliz@ug.uchile.cl; 2 Programa de Doctorado en Ciencias Silvoagropecuarias y Veterinarias, Campus Sur, Universidad de Chile, Santa Rosa 11315, La Pintana, Santiago 8820808, Chile; 3 Laboratory of Veterinary Vaccines, Department of Animal Biology, Faculty of Veterinary and Animal Science, Universidad de Chile, Santiago 8820808, Chile; svidalvilches@gmail.com (V.S.); danisiel@uchile.cl (S.D.); thardman@students.pitzer.edu (H.T.); valefarias95@gmail.com (F.V.); 4 Núcleo de Investigaciones Aplicadas en Ciencias Veterinarias y Agronómicas, Facultad de Medicina Veterinaria y Agronomía, Campus Maipú-Sede, Universidad de las Américas, Santiago 9250000, Chile; mguzmanm@udla.cl; 5 Department of Preventive Medicine, Faculty of Veterinary and Animal Science, Universidad de Chile, Santiago 8820808, Chile; llapierre@uchile.cl

**Keywords:** canine parvovirus, immune escape, antigenic drift, full length VP2, molecular characterization, selection pressures

## Abstract

**Simple Summary:**

Canine parvovirus (CPV) is a relevant pathogen, mainly affecting unvaccinated puppies, causing severe and fatal disease. CPV is classified into three variants (CPV-2a, CPV-2b and CPV-2c), which are widely distributed worldwide. These variants may be mutated at specific sites relevant to the immune response against CPV in dogs, and thus previously characterized vaccines may not be effective against new mutants. Therefore, the aim of the present study was to perform a molecular characterization of CPV variants. For this purpose, blood samples from canine patients in central Chile were used. The results of this study showed that the circulating variants were mainly CPV-2c followed by CPV-2b. In addition, genetic mutations were found in regions important for the immune response against CPV, which possibly has implications for the protective immunity generated by available vaccines.

**Abstract:**

Canine parvovirus (CPV) is a major pathogen in canines, with a high mortality rate in unvaccinated puppies. CPV is traditionally classified into three antigenic variants (CPV-2a, CPV-2b and CPV-2c) based on the amino acid sequence of the VP2 protein. Currently, various mutations are described in the receptor-binding area or in the regions of greatest antigenicity of the VP2 protein, giving rise to new viral variants that are capable of immunological escape, affecting the protective immunity of traditional vaccines. In the present study, a molecular characterization of the VP2 gene was performed, which included phylogenetic analysis, amino acid characterization and determination of selection pressures. Blood samples were initially collected from canine patients with clinical signs of gastrointestinal infection, of which 69 were positive for CPV as measured by means of PCR and 18 samples were selected for the amplification of the complete VP2 gene. The analysis revealed a higher rate of CPV-2c-positive patients compared to CPV-2b. Furthermore, the amino acid characterization of VP2 indicated mutations in the regions of highest antigenicity previously described in the literature (CPV-2b: 297 and 324; CPV-2c: 440), as well as others not previously documented (CPV-2b: 514; CPV-2c: 188, 322, 379, 427 and 463). Our analysis of selection pressure showed that the VP2 gene is under negative selection. However, positive selection point sites were identified, both in CPV-2c (324, 426 and 440) and CPV-2b (297 and 324), at sites that have been associated with evasion of the immune response via antigenic drift, which possibly has implications for the protective immunity generated by traditional vaccines.

## 1. Introduction

Canine parvovirus (CPV) belongs to the *Protoparvovirus* genus and is a part of the *Carnivore protoparvovirus 1* species [1]. Its genome is comprised of 5200 nucleotides of single-stranded DNA (ssDNA) and includes two open reading frames (ORFs). The first encodes for non-structural proteins (NS1 and NS2) that participate in DNA replication, viral transport and capsid assembly [2], and the second ORF encodes the capsid structural proteins “VP1 and VP2”. The VP2 protein has 584 amino acid residues and is the most abundant and immunogenic, fulfilling a key role in viral tropism and host range [3,4,5]. CPV-2 was first described in 1978 as a consequence of a change in a few amino acids in the VP2 protein that allowed the interspecies jump from other carnivores to dogs [6]. This virus spread rapidly worldwide causing a pandemic characterized by symptoms such as hemorrhagic diarrhea, gastroenteritis, vomiting and immunosuppression [7]. Later, in 1979, CPV-2a was discovered, which differs only by four amino acids from the VP2 protein (M87L, I101T, A300G, D305Y). In 1984 in the United States and then in 2000 in Italy, variants 2b and 2c emerged, respectively [8,9]. These antigenic variants were classified by changes in amino acid 426 (CPV-2a: N; CPV-2b: D; and CPV-2c: E) and are currently distributed around the world [8,9]. In South American countries, such as Argentina, Uruguay, Brazil, Ecuador, Peru and Chile, a prevalence of CPV-2c over viral variants CPV-2a and CPV-2b has been documented [10,11,12,13,14,15,16]. The presence of new mutations in specific amino acids of VP2 (267, 297, 324 and 440) located in high antigenicity regions and in sites associated with transferrin receptor binding have been described [17,18,19,20,21], giving rise to new viral variants [22,23]. One of the main characteristics of these mutations is based on the enhancement of immune escape of CPV, via antigenic drift. This seems to have contributed to reduced efficacy of the commonly used vaccines, which are generally comprised of the original variant of the CPV-2 or CPV-2b virus, calling into question the generation of cross immunity [22,23]. Decreased efficacy has been evidenced through studies showing animals with up-to-date vaccinations being sick [10,18,24]. Many of these new mutations have been subjected to positive selection processes [17,20,25,26,27], expressing themselves more frequently across generations and favoring the optimization of viral fitness and evasion of the host immune response generated mainly by traditional vaccines [17,28]. Taking into account the wide molecular diversity and adaptability of CPV, the objective of this study was the identification of viral variants from the complete VP2 gene in the central zone of Chile. Similarly, amino acid substitutions and selection pressures in VP2 were studied in order to evaluate the phenotypic changes associated with evasion of the immune response.

## 2. Materials and Methods

### 2.1. Clinical Sample Collection

A total of 100 blood samples were collected from canine patients between 2 and 108 months of age from different areas of the Metropolitan Region of Chile (central, south, north, east and west) between January and April of the year 2019. The samples were obtained from patients showing clinical symptoms of gastrointestinal infectious disease, such as vomiting, hemorrhagic diarrhea, dehydration, decay, anorexia and fever. Blood samples were collected in tubes with anticoagulant (Becton and Dickinson Vacutainer, Franklin Lakes, NJ, USA) and refrigerated (2–8 °C) until further analysis.

Animal management and maintenance protocols were approved by the Bioethics Committee at the Faculty of Animal and Veterinary Sciences, University of Chile (certificate n 20396 VET-UCH). Each owner previously signed an informed consent agreeing to the entry of their pet into the study.

### 2.2. DNA Extraction and Partial Amplification of VP2 Gene

DNA was extracted from 200 μL of blood sample, using the Exgene Cell SV kit (GeneAll), according to the manufacturer’s recommendations. Conventional PCR was performed using the Taq polymerase (RBC) and the VP2-F (5′-CAGGAAGATATCCAGAAGGA-3′) and VP2-R (5′-GGTGCTAGTTGATATGTAATAAACA-3′) primers, which allowed amplifying a total of 719 bp, spanning the region from 467 bp to 1165 bp of the gene encoding the canine parvovirus VP2 protein [9]. The amplicons were then visualized by staining DNA GelRed^®^ (20,000× per solution, Biotium Inc., Fremont, CA, USA) on 2% agarose gel. DNA from the commercial Novibac^®^ Puppy DP vaccine (Merck Sharp & Dohme Animal Health, Boxmeer, The Netherlands) was used as a positive control.

### 2.3. Sequencing

PCR was carried out using the Q5^®^ High Fidelity DNA polymerase (New England Biolabs, MA, USA) in samples that were positive for CPV, with the cVP2F (5′-GGTGCAGGAGGACAAGTAAAAAGAG-3′) and cVP2R (5′-ACCCACACCATAACAACATACA-3′) primers to amplify the complete sequence of the VP2 protein [15]. The amplicons were visualized under the previously described conditions. Samples that were properly amplified were selected to be purified with the DNA Clean & Concentrator^®^ kit (Zymo Research, Irvine, CA, USA) and sent to Macrogen (Seoul, Korea) for sequencing.

### 2.4. Dataset

The chromatograms of the sequenced samples were evaluated with the FinchTV.V4 software. The nucleotide sequences were then aligned with the ClustalX 2.1 program, including the reference gene for the canine parvovirus VP2 protein (GenBank ID: M38245) to ensure the alignments were within the correct framework. In addition, a dataset was generated that included the study samples (2P-CL, 5P-CL, 9P-CL, 11P-CL, 13P-CL, 16P-CL, 18P-CL, 22P-CL, 26P-CL, 27P-CL, 29P-CL, 33P-CL, 35P-CL, 49P-CL, 56P-CL, 60P-CL, 83P-CL and 87P-CL), the reference gene for feline panleukopenia virus VP2 protein (FPV), VP2 genes from commercial vaccines as well as samples from different parts of the world, including the VP2 reference genes from South America (Argentina, Uruguay, Brazil and Ecuador), North America (United States), Europe (Germany, Italy, Spain, France and Portugal), Asia (China, South Korea and Taiwan) and Africa (Nigeria). All GenBank access numbers are indicated in Supplementary Table S1.

### 2.5. Phylogenetic Analysis

From the samples included in the dataset, a maximum likelihood (ML) phylogenetic tree was inferred with 1000 bootstraps to support the nodes, using the PhyML 3.0 platform, available online: http: //www.atgc-montpellier.fr/phyml, (accessed on 1 May 2021) [29], selecting the HKY + I + G nucleotide substitution model according to the Akaike (AIC) and Bayesian information criteria, implemented in Jmodeltest [30]. In turn, the phylogenetic tree was represented with the Interactive Tree of Life software [31].

### 2.6. Amino Acid Analysis of VP2

Total nucleotide sequences were translated into amino acids to evaluate the conserved and variable sites along the VP2 protein using the MEGA-X software V10.0.05. Antigenic classification of the virus into CPV-2a, CPV-2b and CPV-2c was based on the substitutions at sites 81, 101, 300, 305 and 426, as well as the identification of alternative substitutions at different sites of the VP2 protein [23]. The Phire² online platform (http://www.sbg.bio.ic.ac.uk/phyre2/html/page.cgi?id=index, accessed on 10 May 2021) [32] was used to model the amino acid substitutions of the viral variants. The amino acids were represented in the Discovery Studio Visualizer 2020 software.

### 2.7. Selection Pressure on the VP2 Protein in Canine Parvovirus 

Non-synonymous substitutions in the VP2 protein result in amino acidic changes with negative, neutral or positive impact in terms of viral fitness and adaptation to its host or environment. Amino acid substitutions with positive selection pressure are characterized by conferring an improvement in viral fitness and may alter its antigenicity, known as antigenic drift, which leads to the fixation of an antigenic variant and eventually evolve and differentiate into different lineages or strains within circulating populations in specific geographic area. Negative selection pressure is characterized by a higher presentation of synonymous substitutions per synonymous site (dS) compared to non-synonymous substitutions per synonymous site (dN); this is associated with a lower presentation of deleterious amino acid substitutions that may affect viral fitness [33]. To evaluate the evolution of CPV-2b and CPV-2c independently, two new sub-datasets were developed that included viral variants of each type, respectively (Supplementary Table S2). For the evaluation of selective pressures at individual sites of codon alignment, the synonymous (dS) and non-synonymous (dN) substitution rates were estimated, where a dN/dS ratio < 1 indicates negative selection and dN/dS ratio > 1 represents positive selection. The analysis was completed using different methods available on the Datamonkey web server (http://www.datamonkey.org, accessed on 25 January 2021), such as: Single-Likelihood Ancestor Counting (SLAC), Fixed Effects Likelihood (FEL), Fast Unconstrained Bayesian Approximation (FUBAR) and Mixed Effects Model of Evolution (MEME) [34].

## 3. Results

### 3.1. Detection and Characterization of Positive Samples for Canine Parvovirus

A CPV-positive animal rate of 69% (n = 69) was obtained from 100 patients with gastrointestinal disease symptomatology by amplifying a partial fragment of the VP2 gene sequence (719 bp). Of the positive patients, 18 samples were successfully sequenced for amplification of the complete VP2 gene (Supplementary Table S3).

### 3.2. Phylogenetic Analysis 

**Figure 1:**
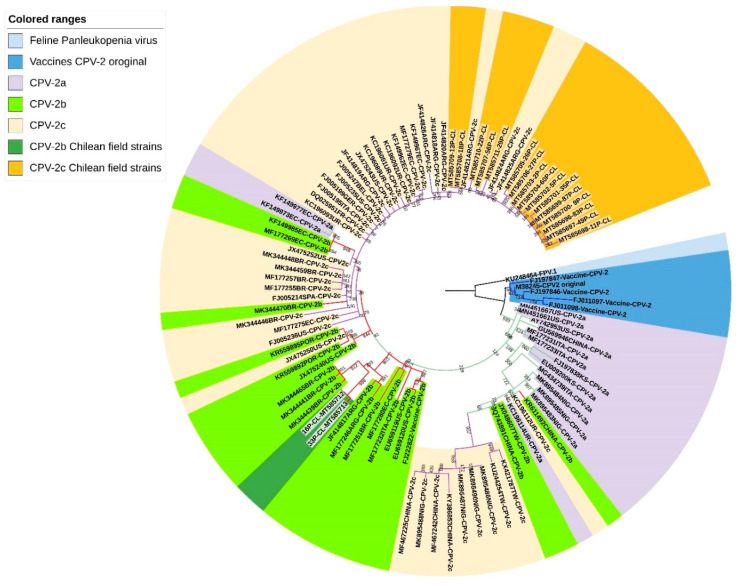
Phylogenetic tree using the maximum likelihood (ML) method. Phylogenetic inferences were carried out using complete nucleotide sequences of the VP2 gene of CPV-2a, CPV-2b and CPV-2c, including different countries in Europe (GER: Germany; ITA: Italy; SPA: Spain; FRA: France; BEL: Belgium; and POR: Portugal), Asia (CHINA; TW: Taiwan; and KS: South Korea), Africa (NIG: Nigeria), North America (US: United States) and South America (ARG: Argentina; UY: Uruguay; EC: Ecuador; BR: Brazil; including the viral variants from Chile: CL). Each sequence was identified according to GenBank accession number, country of origin and viral variant. The tree included as its root an FPV sequence. The blue branches include the variants associated with the reference gene for CVP-2 and commercial vaccines (CVP-2); the green, red and purple branches indicate the viral variants CVP-2a, CVP-2b and CVP-2c, respectively.

From the phylogenetic analysis of the complete VP2 gene, it was inferred that the Chilean samples 16P-CL and 33P-CL are grouped in the same clade with strains described in Argentina (CPV-2b) and Brazil (CPV-2b), suggesting a close phylogenetic relationship. The other 16 Chilean samples are part of a clade composed mainly of CPV-2c viral variants from different parts of the world, being phylogenetically closer to those of Argentina. All the Chilean samples are genetically distant from the commercial vaccines that have the CPV-2 or CPV-2b variant. (Figure 1).

### 3.3. Amino Acid Analysis of the VP2 Protein in Canine Parvovirus 

The amino acid analysis allowed the samples to be classified antigenically into CPV-2b (n = 2) and CPV-2c (n = 16). In parallel, non-synonymous substitutions were identified at sites 188 (n = 1), 297 (n = 2), 322 (n = 1), 324 (n = 2), 379 (n = 1), 427 (n = 1), 440 (n = 16), 463 (n = 1) and 514 (n = 2), compared to the reference strains obtained from GenBank (Table 1).

**Figure 2:**
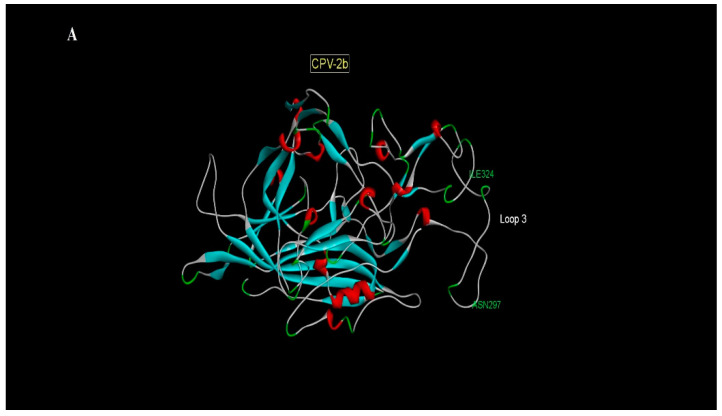

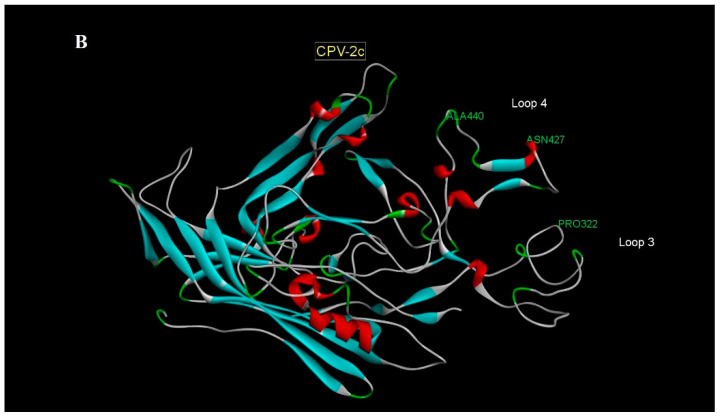
Modeling of the VP2 structure, indicating the substitutions on the triple spike. (**A**) Representation of more relevant substitutions in variant CPV-2b at sites A297N and Y324I; and (**B**) CPV-2c at sites T322P, D427N and T440A. For the modeling of amino acid substitutions, the Phire² platform (http://www.sbg.bio.ic.ac.uk/phyre2/html/page.cgi?id=index, accessed on 10 May 2021) [32] was used and visualized using Discovery Studio Visualizer Server Copyright © 2019.

In the viral variants of the study, substitutions were identified in the barrel β structure (CPV-2b: A516S; CPV-2c: A188S; and V463I), in loop 3 (CPV-2b: A297N and Y324I; CPV-2c: T322P and A379S) and loop 4 of the triple spike (D427N and T440A) (Figure 2).

### 3.4. Selection Pressure Analysis of the Samples in the Study 

Selection pressure analyses carried out from the sequenced samples of the VP2 gene yielded a dN/dS value of 0.0941 and 0.1 for CPV-2c and CPV-2b, respectively. This indicates that the VP2 gene is under negative selection. However, there were specific positive selection sites, both in CPV-2c (324, 426 and 440) and CPV-2b (297 and 324) (Table 2).

## 4. Discussion

CPV is one of the most prevalent pathogens affecting domestic canines, causing high mortality rates in young unvaccinated or immunosuppressed animals [7]. Since the discovery of CPV in the late 1970s, the virus has constantly evolved, leading to the emergence of different viral variants (CPV-2a, CPV-2b and CPV-2c), classified antigenically by the amino acid composition of the VP2 protein, that have spread around the world [8,9]. The current study represents the first molecular, amino acid, and pressure selection characterization of the complete CPV VP2 gene in Chile. Phylogenetic analysis revealed that the Chilean samples are closer to the viral variants in the region. The CPV-2b samples were grouped with viral variants from Argentina and Brazil, and the CPV-2c samples with viral variants originating in Argentina. It has been described at the continental level that the viral variants present in Argentina, Ecuador and Uruguay come from southern Europe and Asia, respectively, and that Argentina has given rise to the most important regional migration, expanding to Uruguay and Brazil [35]. These migration events support the hypothesis that the Chilean samples come from nearby countries, such as Argentina and Brazil, and would explain the groupings evidenced in the phylogenetic tree. Only one molecular identification study of CPV has been completed in Chile, based on the partial amplification of the VP2 gene, which has identified the co-circulation of CPV-2c and CPV-2a, CPV-2c being most prevalent [16]. This result differs from that obtained in the present study, where a predominance of CPV-2c and the presence of CPV-2b was evidenced. A CPV-2a variant was not identified in any of the samples analyzed. This difference could be due to the fact that these studies were carried out at different years and geographical areas of Chile, both of which have been associated with the diversity of viral variants of CPV within the same country [36]. Interestingly, the predominance of CPV-2c observed in Chile, coincides with that described in the rest of the countries of South America [10,11,12,13,14,15,21] and the world [37,38], revealing its success in spread and invasion.

Amino acid characterization studies confirmed various nonsynonymous substitutions at previously described sites (CPV-2b: 297 and 324; CPV-2c: 440), as well as others not previously documented (CPV-2b: 514; CPV-2c: 188, 322, 379, 427 and 463). From a conformational point of view, some of these substitutions (CPV-2b: A514S; CPV-2c: A188S and V463I) are part of the VP2 barrel β structure, whose main function is to provide stability to the capsid structure [3]. These sites are not exposed in the capsid and have a higher degree of structural conservation compared to the triple spike, so the real implication in viral pathogenesis is unknown [37]. However, in countries such as Ecuador and Colombia, CPV-2a variants with substitutions at the A514S site have been identified, suggesting that this mutation could favor stronger binding to the transferrin receptor type I (tfr) receptor or prevent neutralization by antibodies [39]. Mutations were evident in sites that make up the triple spike (loops 1, 2, 3 and 4), at sites not previously described (CPV-2c: 322, 379 and 427), and could be linked to local adaptation processes in response to different patterns of evolutionary pressure [17]. However, it is necessary to carry out future studies that corroborate this approach. Interestingly, amino acid substitutions were evident in the triple spike at sites widely described in the literature in both CPV-2b and CPV-2c. Specifically, in the CPV-2b samples, substitutions were identified at sites A297N and Y324I, which are exposed on the surface of VP2 and are positioned on the triple spike (loop 3) or on sites adjacent to the triple spike, respectively [3]. Particularly, site 297 is located close to residue 300, which together with amino acids 93 and 323 participate in the recognition of the tfr in canines [5]. Substitutions at site 297 have been associated with positive selection processes, contributing fundamentally to viral immune escape [17]. At the same time, substitutions at this S297A site have shaped the new variants of CPV-2a and CPV-2b, which generally include substitutions at other sites, such as, 267, 324 and 440 [21,23]. Furthermore, the A297N substitution found in this study coincides with that identified by researchers from Brazil [17], Colombia [39] and Argentina, making this a unique substitution for samples from South America [18]. Another non-synonymous substitution found in the CPV-2b samples of the study was Y324I, which is adjacent to amino acid 323, which participates in the recognition of tfr type I and plays a fundamental role in the adaptation of the canine host range [5]. The Y324I substitution has been reported in viral variants CPV-2a and CPV-2b [12,19] and it seems to promote stronger binding with the tfr type I receptor [40]. The presence of this mutation in viral variants has been associated with failure in the efficacy of traditional vaccines in previously immunized patients [19].

Mutations in the CPV-2b samples seem to contribute to improved viral fitness, associated with a stronger bond with tfr and the promotion of immune escape [22,23]. To corroborate this, additional studies are required to evaluate these substitutions and their relationship to the pathogenesis of CPV. The T440A substitution was identified in all the CPV-2c samples in the study. This residue is positioned at the tip of the triple spike (loop 4) and is highly exposed on the surface, corresponding to one of the most immunogenic sites of the capsid [3]. The T440A mutation has been identified in different parts of the world, both in the CPV-2a, CPV-2b and CPV-2c variants [10,11], and is one of the characteristic substitutions of the new viral variants of CPV [22,23]. This site is subjected to positive selection, evolving independently in different populations, which explains its presence around the world, not being related to particular variants of CPV-2 [36]. Interestingly, mutations in the T440A site of CPV-2c have been associated with failures in vaccine efficacy, resulting in sickness in animals presenting with up-to-date vaccinations in Argentina [10,18] and India [24]. A similar scenario occurred in the present study, as two patients with CPV-2c with a T440A substitution (29P-CL and 87P-CL) presented a complete vaccination schedule and displayed clinical symptomatology of the disease (data not shown). Together, this background allows us to deduce that the substitution at position T440A favors immune escape against the host’s immune response via antigenic drift and may be responsible for failure to immunize with traditional vaccines.

Finally, the selection pressure analyses performed in this study revealed that the VP2 gene is under negative selection pressure. There were specific positive selection sites, both in CPV-2c (324, 426 and 440) and CPV-2b (297 and 324). The vast majority of these positive selection sites coincide with the results of the amino acid characterization carried out in the present study. This indicates that the sites that are subjected to positive selection pressures worldwide are also present in samples from Chile, assuming that one of the possible causes of this phenomenon is associated with a basal level of immunological protection in the population of canines that promoted the appearance of more suitable viral strains [17]. The only site with positive selection that did not coincide with the amino acid characterization in CPV-2c was residue 324, which was possibly positive due to the inclusion of viral variants from Asia and Africa that include mutations in that site. The fundamental importance of knowing sites with positive selection pressure is based on the fact that they represent beneficial mutations that increase in frequency across generations, optimizing the fitness of the virus while favoring the evasion of the host immune response generated by traditional vaccines [17,28]. The selection pressure results of this study differ from what was obtained by other authors [17,20,25,26,27,37]. These differences are mainly attributed to the composition of the datasets used to infer the selection pressures, since it has been seen that modifications in these can alter the selection pressure results [37]. The amino acid characterization and the selection pressure studies allow us to deduce that the CPV samples identified in this study have been regionally adapted, undergoing positive selection processes and resulting in viral mutations via antigenic drift, both in CPV-2c and CPV-2b, which possibly contribute to evasion of the immune response. Future functional studies are required to determine the implication of these amino acid substitutions in VP2 and their relationship with immune escape.

## 5. Conclusions

This is the first molecular characterization study in Chile that includes the complete VP2 gene in Chile. Our results demonstrate the co-circulation of CPV-2c and CPV-2b in the central zone of the country, with a predominance of CPV-2c. Likewise, through phylogenetic analysis, it is suggested that the Chilean samples possibly come from neighboring countries, such as Argentina and Brazil. In both variants, amino acid substitutions were identified in the regions of highest VP2 antigenicity. Some of these mutations coincidentally were subjected to positive selection processes in sites that have been previously described in the literature and have been associated with evasion of the immune response, via antigenic drift, which possibly has implications for the protective immunity generated by traditional vaccines.

## Figures and Tables

**Table 1:** Amino acid analysis of the VP2 protein in canine parvovirus.

Viral Variant	87 *	101 *	188	297	300 *	305 *	322	324	379	426 *	427	440	463	514
CPV-2 original (M38245.1)	M	I	A	S	A	D	T	Y	A	N	D	T	V	A
Vaccine-Merial (FJ01197.1)	·	·	·	·	·	·	·	·	·	·	·	·	·	·
Vaccine-Intervet (FJ01198.1)	·	·	·	·	·	·	·	·	·	·	·	·	·	·
Vaccine-CPV-Intervet (FJ197846.1)	·	·	·	·	·	·	·	·	·	·	·	·	·	·
Vaccine-Pfizer (FJ197847.1)	·	·	·	·	·	·	·	·	·	·	·	·	·	·
CPV-2a (MF177233)	L	T	A	A	G	Y	T	Y	A	N	D	T	V	A
CPV-2b (EU659119) USA	L	T	A	A (GCT)	G	Y	T	Y (TAT)	A	D	D	T	V	A
														(GCA)
Vaccine Duramune (FJ222822.1)	·	·	·	·	·	·	·	·	·	·	·	·	·	·
Field sample: 16P-CL	·	·	·	N (AAT)	·	·	·	I (ATT)	·	·	·	·	·	S (TCA)
Field sample: 33P-CL	·	·	·	N (AAT)	·	·	·	I (ATT)	·	·	·	·	·	S (TCA)
CPV-2c (MK344446) Brasil	L	T	A (GCA)	A	G	Y	T (ACA)	Y	A	E	D (GAT)	T	V (GTT)	A
									(GCA)			(ACA)		
Field sample: 2P-CL	·	·	·	·	·	·	·	·	·	·	·	A (GCA)	·	·
Field sample: 5P-CL	·	·	·	·	·	·	·	·	·	·	·	A (GCA)	·	·
Field sample: 9P-CL	·	·	·	·	·	·	·	·	·	·	·	A (GCA)	·	·
Field sample: 11P-CL	·	·	·	·	·	·	·	·	·	·	N (AAT)	A (GCA)	·	·
Field sample: 13P-CL	·	·	·	·	·	·	·	·	·	·	·	A (GCA)	·	·
Field sample: 18P-CL	·	·	·	·	·	·	·	·	·	·	·	A (GCA)	·	·
Field sample: 22P-CL	·	·	·	·	·	·	P (CCA)	·	·	·	·	A (GCA)	·	·
Field sample: 26P-CL	·	·	·	·	·	·	·	·	S (TCA)	·	·	A (GCA)	·	·
Field sample: 27P-CL	·	·	·	·	·	·	·	·	S (TCA)	·	·	A (GCA)	·	·
Field sample: 29P-CL	·	·	S (TCA)	·	·	·	·	·	·	·	·	A (GCA)	·	·
Field sample: 35P-CL	·	·	·	·	·	·	·	·	·	·	·	A (GCA)	·	·
Field sample: 49P-CL	·	·	·	·	·	·	·	·	·	·	·	A (GCA)	·	·
Field sample: 56P-CL	·	·	·	·	·	·	·	·	·	·	·	A (GCA)	I (ATT)	·
Field sample: 60P-CL	·	·	·	·	·	·	·	·	·	·	·	A (GCA)	·	·
Field sample: 83P-CL	·	·	·	·	·	·	·	·	·	·	·	A (GCA)	·	·
Field sample: 87P-CL	·	·	·	·	·	·	·	·	·	·	·	A (GCA)	·	·

* Amino acid sequences of the original CPV-2 variant.

**Table 2:** Selection pressure on VP2 in the sequenced samples.

	Selection Pressures on the Genome of the VP2 Canine Parvovirus							
	Sites of Positive Selection				Sites of Negative Selection			
Viral Variant	SLAC ¹	FEL ^1^	FUBAR ^2^	MEME ¹	SLAC ^1^	FEL ^1^	FUBAR ^2^	Mean dN/dS
CPV-2c	Non	Non	426, 440	324	Negative selection in 1 site	Negative selection in 31 sites	Negative selection in 50 sites	0.0941
CPV-2b	Non	Non	324	297, 324	Non	Negative selection in 23 sites	Negative selection in 41 sites	0.1

^1^*p*-value < 0.05; ^2^ posterior probability ≥ 0.90.

## Data Availability

The sequences generated in this study are available in GenBank under.

## Supplementary Materials

The following are available online at https://www.mdpi.com/article/10.3390/ani11082387/s1, Table S1: Number of GenBank accesses and geographical location of the different samples included in the dataset; Table S2: Number of GenBank accesses and geographical location of the different samples included for the CPV-2b and CPV-2c sub-datasets. Table S3: General history of canines in study.
