# Supplementary material for: Molecular Analysis of Full-Length VP2 of Canine Parvovirus Reveals Antigenic Drift in CPV-2b and CPV-2c Variants in Central Chile
Source: Animals (Basel). 2021 Aug 12;11(8):2387. doi: 10.3390/ani11082387 (PMC8388783; doi:10.3390/ani11082387)
Supplement: Supplementary file 1 [file animals-11-02387-s001.zip › animals-1276397-supplementary.pdf]

Supplementary Table S1. Number of GenBank accesses and geographical location of the different samples included in the dataset.

Number	Virus type	Country	GenBank Accesion number	Number	Virus type	Country	GenBank Accesion number
1	FPV	N.A.	KU248464	41	CPV-2c	Ecuador	MF177275
2	Vaccine CPV-2	N.A.	FJ197846	42	CPV-2c	Ecuador	MF177279
3	Vaccine CPV-2	N.A.	FJ197847	43	CPV-2a	USA	MN451661
4	Vaccine CPV-2	N.A.	FJ011097	44	CPV-2a	USA	MN451667
5	Vaccine CPV-2	N.A.	FJ011098	45	CPV-2a	USA	AY742953
6	Vaccine CPV-2b	N.A.	FJ222822	46	CPV-2b	USA	JX47524
7	CPV-2b	Argentina	JF414817	47	CPV-2c	USA	JX475243
8	CPV-2b	Argentina	MF177246	48	CPV-2c	USA	FJ005235
9	CPV-2c	Argentina	JF414818	49	CPV-2c	USA	FJ005236
10	CPV-2c	Argentina	JF414819	50	CPV-2c	USA	JX475250
11	CPV-2c	Argentina	JF414820	51	CPV-2c	USA	JX475252
12	CPV-2c	Argentina	JF414821	52	CPV-2c	Germany	FJ005199
13	CPV-2c	Argentina	JF414824	53	CPV-2a	Italy	MG434739
14	CPV-2c	Argentina	JF414825	54	CPV-2a	Italy	MF177231
15	CPV-2c	Argentina	JF414826	55	CPV-2a	Italy	MF177233
16	CPV-2a	Uruguay	KC196114	56	CPV-2b	Italy	MF177232
17	CPV-2c	Uruguay	KC196112	57	CPV-2c	Italy	FJ005195
18	CPV-2c	Uruguay	KC196089	58	CPV-2c	Spain	FJ005214
19	CPV-2c	Uruguay	KC196092	59	CPV-2c	France	DQ025951
20	CPV-2c	Uruguay	KC196093	60	CPV-2c	Belgium	FJ005247
21	CPV-2c	Uruguay	KC196091	61	CPV-2b	Porrtugal	KR559895
22	CPV-2b	Brazil	MF177251	62	CPV-2b	Porrtugal	KR559892
23	CPV-2b	Brazil	EU659119	63	CPV-2a	China	GU569946
24	CPV-2b	Brazil	EU659120	64	CPV-2b	China	KR611492
25	CPV-2b	Brazil	MK344439	65	CPV-2b	China	JQ743891
26	CPV-2b	Brazil	MK344465	66	CPV-2c	China	MF467225
27	CPV-2b	Brazil	MK344441	67	CPV-2c	China	MF467242
28	CPV-2b	Brazil	MK344470	68	CPV-2c	China	KY386853
29	CPV-2c	Brazil	MF177257	69	CPV-2a	South Korea	EU009200
30	CPV-2c	Brazil	MK344459	70	CPV-2a	South Korea	FJ197838
31	CPV-2c	Brazil	MK344446	71	CPV-2b	Taiwan	JX048607
32	CPV-2c	Brazil	MK344448	72	CPV-2c	Taiwan	KU244254
33	CPV-2c	Brazil	MF177255	73	CPV-2c	Taiwan	KX421787
34	CPV-2a	Ecuador	KF149977	74	CPV-2a	Nigeria	MK895483
35	CPV-2a	Ecuador	KF149973	75	CPV-2a	Nigeria	MK895484
36	CPV-2b	Ecuador	MF177280	76	CPV-2a	Nigeria	MK895485
37	CPV-2b	Ecuador	KF149985	77	CPV-2c	Nigeria	MK895488
38	CPV-2b	Ecuador	MF177269	78	CPV-2c	Nigeria	MK895486
39	CPV-2c	Ecuador	KF149962	79	CPV-2c	Nigeria	MK895487

40	CPV-2c	Ecuador	KF149967	80	CPV-2c	Nigeria	MK895490

N.A: does not apply to reference vaccines and feline panleukopenia virus.

Supplementary Table S2. Number of GenBank accesses and geographical location of the different samples included for the CPV-2b and CPV-2c sub-datasets.

Subdataset CPV-2b				Subdataset CPV-2c			
Number/ ID	Virus type	Country	GenBank Accesion number	Number /ID	Virus type	Country	GenBank Accesion number
1	CPV-2b	Argentina	JF414817	1	CPV-2c	Argentina	JF414818
2	CPV-2b	Argentina	MF177246	4	CPV-2c	Argentina	JF414819
3	CPV-2b	Brazil	MF177251	5	CPV-2c	Argentina	JF414820
4	CPV-2b	USA	EU659119	6	CPV-2c	Argentina	JF414821
5	CPV-2b	USA	EU659120	7	CPV-2c	Argentina	JF414824
6	CPV-2b	Brazil	MK344439	8	CPV-2c	Argentina	JF414825
7	CPV-2b	Brazil	MK344465	9	CPV-2c	Argentina	JF414826
8	CPV-2b	Brazil	MK344441	10	CPV-2c	Uruguay	KC196112
9	CPV-2b	Brazil	MK344470	11	CPV-2c	Uruguay	KC196089
10	CPV-2b	Ecuador	MF177280	12	CPV-2c	Uruguay	KC196092
11	CPV-2b	Ecuador	KF149985	13	CPV-2c	Uruguay	KC196093
12	CPV-2b	Ecuador	MF177269	14	CPV-2c	Uruguay	KC196091
13	CPV-2b	USA	JX47524	15	CPV-2c	Brazil	MF177257
14	CPV-2b	Italy	MF177232	16	CPV-2c	Brazil	MK344459
15	CPV-2b	Porrtugal	KR559895	17	CPV-2c	Brazil	MK344446
16	CPV-2b	Porrtugal	KR559892	18	CPV-2c	Brazil	MK344448
17	CPV-2b	China	KR611492	19	CPV-2c	Brazil	MF177255
18	CPV-2b	China	JQ743891	20	CPV-2c	Ecuador	KF149962
19	CPV-2b	Taiwan	JX048607	21	CPV-2c	Ecuador	KF149967
16P-CL	CPV-2b	Chile	MT585712	22	CPV-2c	Ecuador	MF177275
33P-CL	CPV-2b	Chile	MT585713	23	CPV-2c	Ecuador	MF177279
				24	CPV-2c	USA	JX475243
				25	CPV-2c	USA	FJ005235
				26	CPV-2c	USA	FJ005236
				27	CPV-2c	USA	JX475250
				28	CPV-2c	USA	JX475252
				29	CPV-2c	Germany	FJ005199
				30	CPV-2c	Italy	FJ005195
				31	CPV-2c	Spain	FJ005214
				32	CPV-2c	France	DQ025951
				33	CPV-2c	Belgium	FJ005247
				34	CPV-2c	China	MF467225
				35	CPV-2c	China	MF467242
				36	CPV-2c	China	KY386853
				37	CPV-2c	Taiwan	KU244254

38	CPV-2c	Taiwan	KX421787
39	CPV-2c	Nigeria	MK895488
40	CPV-2c	Nigeria	MK895486
41	CPV-2c	Nigeria	MK895487
42	CPV-2c	Nigeria	MK895490
43	2P-CL	Chile	MT585703
44	5P-CL	Chile	MT585702
45	9P-CL	Chile	MT585700
46	11P-CL	Chile	MT585698
47	13P-CL	Chile	MT585709
48	18P-CL	Chile	MT585708
49	22P-CL	Chile	MT585710
50	26P-CL	Chile	MT585705
51	27P-CL	Chile	MT585706
52	29P-CL	Chile	MT585711
53	35P-CL	Chile	MT585701
54	49P-CL	Chile	MT585697
55	56P-CL	Chile	MT585707
56	60P-CL	Chile	MT585704
57	83P-CL	Chile	MT585696
58	87P-CL	Chile	MT585699

Supplementary Table S3. General history of canines in study.

Sample identification	Breed	Age (months)	Vaccination status	Status of CPV	Amplification VP2 (bp)	Antigenic variant CPV-2
1P-CL	Mixed breed	3,5	Unknown	+	719	Undetermined
**2P-CL**	**German Shepherd**	**3**	**Incomplete**	**+**	**1755**	**CPV-2c**
3P-CL	Mixed breed	2	Unknown	+	719	Undetermined
4P-CL	Mixed breed	2	Unknown	+	719	Undetermined
**5P-CL**	**Mixed breed**	**2,5**	**Incomplete**	**+**	**1755**	**CPV-2c**
6P-CL	Mixed breed	12	No vaccination	-	-	Negative
7P-CL	Mixed breed	10	Incomplete	-	-	Negative
8P-CL	Mixed breed	6	No vaccination	-	-	Negative
**9P-CL**	**Mixed breed**	**3**	**No vaccination**	**+**	**1755**	**CPV-2c**
10P-CL	Mixed breed	5	Complete	+	719	Undetermined
**11P-CL**	**Dachshund**	**2**	**No vaccination**	**+**	**1755**	**CPV-2c**
12P-CL	Mixed breed	48	Unknown	-	-	Negative
13P-CL	Mixed breed	12	No vaccination	+	**1755**	**CPV-2c**
14P-CL	Mixed breed	72	Unknown	-	-	Negative
15P-CL	Golden Retriever	2	Unknown	-	-	Negative
**16P-CL**	**Yorkshire Terrier**	**3**	**Unknown**	**+**	**1755**	**CPV-2b**
17P-CL	Mixed breed	5	No vaccination	-	-	Negative
**18P-CL**	**Mixed breed**	**3**	**No vaccination**	**+**	**1755**	**CPV-2c**
19P-CL	Mixed breed	3	No vaccination	+	719	Undetermined
20P-CL	Poodle	2	No vaccination	+	719	Undetermined
21P-CL	Mixed breed	2	Unknown	-	-	Negative
**22P-CL**	**Poodle**	**3**	**Incomplete**	**+**	**1755**	**CPV-2c**
23P-CL	Malinois Shepherd	4	Incomplete	+	719	Undetermined
24P-CL	Mixed breed	108	Incomplete	-	-	Negative

25P-CL	Mixed breed	7	No vaccination	+	719	Undetermined
**26P-CL**	**Dachshund**	**1,5**	**Incomplete**	**+**	**1755**	**CPV-2c**
**27P-CL**	**Basset Hound**	**7**	**Unknown**	**+**	**1755**	**CPV-2c**
28P-CL	Mixed breed	2	No vaccination	+	719	Undetermined
**29P-CL**	**Mixed breed**	**4**	**Complete**	**+**	**1755**	**CPV-2c**
30P-CL	Mixed breed	3	No vaccination	-	-	Negative
31P-CL	Mixed breed	5	Unknown	-	-	Negative
32P-CL	Mixed breed	4	No vaccination	+	719	Undetermined
**33P-CL**	**Mixed breed**	**6**	**Incomplete**	**+**	**1755**	**CPV-2b**
34P-CL	Mixed breed	6	Unknown	-	-	Negative
**35P-CL**	**Mixed breed**	**3**	**Unknown**	**+**	**1755**	**CPV-2c**
36P-CL	Mixed breed	2	Unknown	+	719	Undetermined
37P-CL	Mixed breed	2	Unknown	-	-	Negative
38P-CL	Mixed breed	2	Unknown	+	719	Undetermined
39P-CL	Mixed breed	2	Unknown	-	-	Negative
40P-CL	Mixed breed	2	Incomplete	+	719	Undetermined
41P-CL	Mixed breed	2	No vaccination	-	-	Negative
42P-CL	Mixed breed	2	No vaccination	-	-	Negative
43P-CL	Mixed breed	3	No vaccination	-	-	Negative
44P-CL	Mixed breed	18	No vaccination	-	-	Negative
45P-CL	Mixed breed	3	No vaccination	+	719	Undetermined
46P-CL	Mixed breed	12	Incomplete	-	-	Negative
47P-CL	German Shepherd	3	No vaccination	+	719	Undetermined
48P-CL	Mixed breed	96	No vaccination	-	-	Negative
**49P-CL**	**Labrador Retriever**	**4**	**Unknown**	**+**	**1755**	**CPV-2c**
50P-CL	Mixed breed	9	No vaccination	+	719	Undetermined
51P-CL	Mixed breed	5	No vaccination	+	719	Undetermined
52P-CL	Yorkshire Terrier	5	Complete	-	-	Negative
53P-CL	Mixed breed	2	No vaccination	+	719	Undetermined

54P-CL	Mixed breed	2	No vaccination	+	719	Undetermined
55P-CL	Rottweiler	7	Unknown	+	719	Undetermined
**56P-CL**	**Mixed breed**	**14**	**Incomplete**	**+**	**1755**	**CPV-2c**
57P-CL	Bullterrier	2	No vaccination	+	719	Undetermined
58P-CL	Pitbull	3	Incomplete	+	719	Undetermined
59P-CL	Mixed breed	2	Unknown	-	-	Negative
**60P-CL**	**Mixed breed**	**4**	**Incomplete**	**+**	**1755**	**CPV-2c**
61P-CL	Yorkshire Terrier	12	Unknown	-	-	Negative
62P-CL	Mixed breed	2	No vaccination	-	-	Negative
63P-CL	Mixed breed	10	Unknown	-	-	Negative
64P-CL	Mixed breed	2	Unknown	+	719	Undetermined
65P-CL	Golden Retriever	3	Incomplete	+	719	Undetermined
66P-CL	Golden Retriever	5	Incomplete	+	719	Undetermined
67P-CL	Chihuahua	2	No vaccination	+	719	Undetermined
68P-CL	German Shepherd	4	No vaccination	+	719	Undetermined
69P-CL	Bullterrier	6	No vaccination	+	719	Undetermined
70P-CL	Mixed breed	2	Unknown	+	719	Undetermined
71P-CL	Mixed breed	2	No vaccination	+	719	Undetermined
72P-CL	Mixed breed	8	No vaccination	-	-	Negative
73P-CL	Mixed breed	4	Unknown	+	719	Undetermined
74P-CL	Mixed breed	24	No vaccination	+	719	Undetermined
75P-CL	Dachshund	3	Complete	+	719	Undetermined
76P-CL	Rottweiler	4	Complete	-	-	Negative
77P-CL	Dachshund	2	Incomplete	+	719	Undetermined
78P-CL	Mixed breed	3	No vaccination	-	-	Negative
79P-CL	Mixed breed	3	Incomplete	+	719	Undetermined
80P-CL	Mixed breed	2	Unknown	+	719	Undetermined
81P-CL	Chilean Fox Terrier	7	Incomplete	+	719	Undetermined
82P-CL	Yorkshire Terrier	2	Unknown	-	-	Negative
**83P-CL**	**Mixed breed**	**6**	**Unknown**	**+**	**1755**	**CPV-2c**

84P-CL	Mixed breed	7	No vaccination	+	719	Undetermined
85P-CL	Mixed breed	2	No vaccination	+	719	Undetermined
86P-CL	Mixed breed	4	Incomplete	-	-	Negative
**87P-CL**	**Siberian Husky**	**3**	**Complete**	**+**	**1755**	**CPV-2c**
88P-CL	Mixed breed	5	Incomplete	+	719	Undetermined
89P-CL	Mixed breed	7	Incomplete	+	719	Undetermined
90P-CL	Mixed breed	6	No vaccination	+	719	Undetermined
91P-CL	Mixed breed	2	No vaccination	+	719	Undetermined
92P-CL	Chilean Fox Terrier	9	No vaccination	+	719	Undetermined
93P-CL	Mixed breed	5	No vaccination	+	719	Undetermined
94P-CL	Border Collie	2	Incomplete	+	719	Undetermined
95P-CL	Mixed breed	2	Incomplete	+	719	Undetermined
96P-CL	Akita	2	No vaccination	-	-	Negative
97P-CL	Yorkshire Terrier	4	Incomplete	+	719	Undetermined
98P-CL	Mixed breed	4	No vaccination	+	719	Undetermined
99P-CL	Mixed breed	11	Unknown	-	-	Negative
100P-CL	Mixed breed	5	Incomplete	+	719	Undetermined
